# Genome-Wide Analysis of *KCS* Gene Family in *Ginkgo biloba* L. and Functional Identification of *KCS7* in Oleic Acid Synthesis

**DOI:** 10.3390/genes16070773

**Published:** 2025-06-30

**Authors:** Xingyu Zhang, Kaifang Fan, Zhi Feng, Zhi Yao, Jinyuan Li, Shuguang Zhang, Xiaoqin Mi, Fuwen Wu, Yiqiang Wang, Meng Li

**Affiliations:** 1 Key Laboratory of Forestry Biotechnology of Hunan Province, Central South University of Forestry and Technology, Changsha 410004, China; zhangxingyu0514@163.com (X.Z.); 15155473558@163.com (K.F.); biotechnologyfeng@163.com (Z.F.); yaosensei@163.com (Z.Y.); 13283719075@163.com (F.W.); 2 Yuelushan Laboratory Carbon Sinks Forests Variety Innovation Center, Central South University of Forestry and Technology, Changsha 410012, China; 3 Administration Bureau of Swan Mountain National Forest Park, Zixing 423406, China; 15243589411@163.com (J.L.); 13707350868@163.com (S.Z.); 4 Xiangxi Autonomous Prefecture Forest Ecological Experimental Station of Hunan Province, Jishou 416000, China; 13707439151@163.com

**Keywords:** *Ginkgo biloba* L., β-ketoacyl-CoA synthase (KCS), oleic acid, transgenic Arabidopsis

## Abstract

**Background:** β-ketolipoyl coenzyme A synthase (KCS) is an essential limiting catalyst involved in carbon chain elongation during fatty acid biosynthesis, characterized by strict substrate specificity. C18:1 (oleic acid) plays a vital role in cell membranes and is essential for nutrient storage and stress defense. There are indications of significant accumulation and rapid synthesis of C18:1 during the early growth stages of *Ginkgo biloba* L. episperm. The *KCS* gene family in *G. biloba* has yet to be analyzed, and the role of *KCS* in oleic acid synthesis remains unexplored. **Methods**: In this study, this issue was investigated using transcriptomic and metabolomic data, bioinformatics analysis to screen a key gene from the *KCS* gene family, and dual validation using yeast and Arabidopsis thaliana expression systems to probe its function. **Results**: A total of 11 members of the *GbKCS* gene family were identified, and the dynamics of these genes were analyzed during exocarp development in the *G. biloba* genome. Among them, the gene designated *GbKCS7* showed a highly direct association with the content of C18:1. Heterologous expression of *GbKCS7* in yeast increased C18:1N12 and C18:1 content by 3.18-fold and 2.07-fold, respectively. Overexpression of *GbKCS7* in Arabidopsis showed that C18:1 was increased by 27.70% and 31.43% in *GbKCS7-OE-1* and *GbKCS7-OE-2* strains, correspondingly, in juxtaposition to the non-transgenic plants. In addition, the content of VLCFAs increased to varying degrees. **Conclusions**: These outcomes offer important insights for investigating the role of *KCS* genes in fatty acid synthesis to further improve *G.biloba* resistance.

## 1. Introduction

Land plants are susceptible to harsh conditions, such as water scarcity, salinity, and extreme temperatures, as well as pests and pathogens during growth and development. Fatty acids and their derivatives have attracted considerable attention due to their extensive involvement in the defense pathways of various plant species [1,2]. Among fatty acids, C16 and C18 fatty acids were not only core intermediates in lipid synthesis but also important antecedents engaged in the synthesis of very-long-chain fatty acids (VLCFAs), such as plant epidermal cuticle substances and waxes. Additionally, they were crucial for minimizing non-stomatal transpiration and conferring tolerance against environmental stresses, including drought, low temperatures, and UV exposure, alongside biotic challenges like pest and pathogen attacks [3,4,5,6].

A common unsaturated fatty acid in plants, C18:1 (oleic acid), served as an energy reserve component and precursor of bioactive molecules (mainly jasmonic acid and jasmonic acid methyl ester) and extracellular barriers (cutin and suberin), plying an important role in plant growth, development, and defense processes [7,8]. In *Arabidopsis thaliana*, C18:1 was extensively involved in defense signaling pathways mediated by salicylic acid (SA) and jasmonic acid (JA) [9]. Notably, loss-of-function mutations in SSI2, which encoded the predominant stearoyl-ACP desaturase (SACPD) isoform, caused a significant reduction in C18:1 level. These metabolic changes induced developmental defects, including stunted growth and spontaneous necrotic lesions in Arabidopsis, while concurrently suppressing jasmonate signaling and compromising disease resistance against fungal pathogens, including B. cinerea [10,11]. In Arabidopsis, C18:1 was found to stimulate phospholipase D (PLD) activity, initiating programmed cell death through H_2_O_2_ signaling while concurrently enhancing tolerance to abiotic stresses [12].

Plant fatty acid biosynthesis occurred through two distinct phases: (1) de novo synthesis of C16 and C18 fatty acids catalyzed by the fatty acid synthase (FAS) complex, followed by (2) elongation to VLCFAs (C > 18) mediated by the endoplasmic reticulum-localized fatty acid elongase (FAE) complex [13,14]. The fatty acid elongase (FAE) complex comprised four enzymatic components: β-ketoacyl-CoA synthase (KCS), β-ketoacyl-CoA reductase (KCR), β-hydroxyacyl-CoA dehydratase (HCD), and enoyl-CoA reductase (ECR). These enzymes sequentially mediated the four-step elongation cycle involving condensation, reduction, dehydration, and final reduction reactions during fatty acid chain extension [15,16]. Exhibiting precise substrate selectivity, KCS served as the pivotal rate-controlling enzyme during fatty acid chain elongation. The final fatty acid profile, including chain length and saturation pattern, was principally regulated by the enzyme’s catalytic properties and substrate binding affinity [17,18,19]. There was a fine division in the *KCS* gene family. Studies in Arabidopsis revealed that while *AtKCS1* and *AtKCS11* demonstrated catalytic competence toward both saturated and mono-unsaturated C16–C24 acyl-CoA substrates, *AtKCS17* displayed preferential activity for saturated C16–C22 acyl-CoA species [20]. *AtKCS1* expression in heterologous systems (yeast and *Nicotiana benthamiana*) elongated C16 fatty acids into C18–C22 derivatives [21]. Moreover, *AtKCS2* and *AtKCS20* mainly synthesized C22 and C24 and *AtKCS5* and *AtKCS6* mainly synthesized C24–C28, while *FAE1*/*AtKCS18* preferentially catalyzed C20/C22 production [22,23].

An ancient relict gymnosperm plant, *G.biloba* L. (*G. biloba*) is renowned for its significant medicinal, nutritional, and ornamental value [24,25,26,27]. Known for its high resistance to various stresses, *G. biloba* demonstrates remarkable adaptability to diverse environments and has a long lifespan. *G. biloba* seeds comprise four parts: the fleshy outer seed coat (episperm), bony middle seed coat, membranous inner seed coat, and kernel. As it matures, the surface of the episperm becomes coated with a hydrophobic white waxy substance [28]. The fleshy sarcocarp has an important protective function for *G. biloba* seeds before the embryo is fully developed [29]. Therefore, it is generally believed that the episperm of *G. biloba* is an important protective tissue. Through the pre-laboratory analysis of metabolomic data from exocarp stages—EP1 (early stage of exocarp differentiation), EP2 (rapid growth stage of exocarp), and EP3 (mature stage of exocarp)—we observed that oleic acid was rapidly synthesized and accumulated during the early developmental stage of the exocarp, particularly in the C18:1 metabolite trend. β-ketoacyl-CoA synthase is crucial for fatty acid production; however, research on the *KCS* gene family remains lacking, and *GbKCS* functions remain unexplored in *G. biloba*. Our understanding of this enzyme’s role in oleic acid synthesis was also limited. To address these gaps, we characterized the *GbKCS* gene family and identified *GbKCS7* through transcriptomic analysis as a key gene associated with C18:1 fatty acid production. Subsequent functional validation in yeast and Arabidopsis systems confirmed its ability to enhance oleic acid accumulation, demonstrating its biological significance in plant lipid metabolism.

## 2. Materials and Methods

### 2.1. Systematic Analysis of GbKCS Genes in G. biloba Genome

Protein homology comparisons were performed using the BLASTp program in TBtools software to identify the *KCS* gene in the genome downloaded from the *G. biloba* Genome Database (http://gigadb.org/dataset/100209, accessed on 6 December 2023) [30]. The NCBI-CDD (https://www.ncbi.nlm.nih.gov/cdd, accessed on 6 December 2023) and Pfam (http://pfam.xfam.org/, accessed on 6 December 2023) databases were used to search for conserved structural domains of the resulting *KCS* to ensure data accuracy. Prior transcriptome and metabolomics data of *G. biloba* episperm were used to screen *KCS* genes showing varied expression during *G. biloba* episperm development [31]. Then, the submitted KCS protein sequences were analyzed using PROTPARAM (https://web.expasy.org/protparam/, accessed on 17 December 2023) for the isoelectric point, instability coefficient, hydrophilic index, and molecular weight to analyze their physicochemical properties. The subcellular localization of GbKCS proteins was predicted using CELLO software (http://cello.life.nctu.edu.tw/, accessed on 17 December 2023).

### 2.2. Mutiple Sequence Alignment and Phylogenetic Analysis

We performed multiple sequence alignments between AtKCS protein sequences, obtained from the Arabidopsis Information Repository (TAIR) database (https://www.arabidopsis.org/, accessed on 21 December 2023), and GbKCS proteins using the Clustal Omega online program (https://www.ebi.ac.uk/Tools/msa/clustalo/, accessed on 21 December 2023). The alignment results were subsequently visualized and displayed using Jalview software (Jalview version 2.10.5). The apple (Malus × *domestica* Borkh.) MdKCS protein sequences were downloaded from the apple genome database (https://iris.angers.inra.fr/gddh13/the-apple-genome-downloads.html, accessed on 21 December 2023) [32]. To study the phylogenetic relationship of GbKCS protein, the evolutionary tree was constructed by MEGA 11.0 using 21 Arabidopsis AtKCS, 28 apple (Malus × domestica) MdKCS, and 11 GbKCS protein sequences. The parameter settings were as follows: phylogeny: maximum likelihood (ML) tree, test of phylogeny: bootstrap method, bootstrap replication: 1000. The phylogenetic results were beautified using the online website ITOL (https://itol.embl.de/, accessed on 21 December 2023).

### 2.3. Chromosomal Location, Conserved Motifs and Gene Structure Analysis

The structure of the *GbKCS* was determined using the gff3 annotation file from *G. biloba* transcriptome data, and its chromosomal location was mapped with the MapGene2 Chromosome (MG2C v2.1) (http://mg2c.iask.in/mg2c_v2.0/, accessed on 25 December 2023). The gene structure was visualized using the Gene Structure Display Server (GSDS 2.0) (http://gsds.gao-lab.org/, accessed on 25 December 2023), while the conserved motifs of protein sequences were predicted and analyzed using the MEME (https://meme-suite.org/meme/tools/meme, accessed on 25 December 2023) online program. Default settings were applied, and the resulting XML format file was submitted to TBtools software for the visualization of conserved motifs [30].

### 2.4. Prediction of Cis-Acting Elements and Expression Analyis of GbKCS Genes

The upstream 2000 bp sequences of all the *GbKCS* genes were extracted from the genomic DNA sequence and submitted to PlantCARE (http://bioinformatics.psb.ugent.be/webtools/plantcare/html/, accessed on 5 January 2024) to predict the potential cis-acting elements in the promoter region, and the cis-acting elements were analyzed using the TBtools software [33]. Additionally, the number, function, and type of cis-acting elements in *GbKCS* genes were compiled. FPKM-based heatmaps by TBtools software displayed *GbKCS* expression dynamics in *G. biloba* episperm. Correlation analysis was performed between the content of C18:1 and the expression levels of *GbKCS* genes, joining analysis of transcriptome-metabolome data.

### 2.5. RNA Extraction and Quantitative Real-Time PCR (qRT-PCR) Analysis

To confirm the confidence of the transcriptome data, we designed primers for all *GbKCS* family members, normalized the data between samples using *GbGAPDH* as a housekeeping gene, and detected these genes by qRT-PCR [34]. Primer sequences were presented in the Supplementary Material (Table S1). RNA was extracted and reverse-transcribed using the Plant RNA Kit (OMEGA, Norcross, GA, USA) and RevertAid First Strand cDNA Synthesis Kit (Thermo Scientific, Waltham, MA, USA), respectively. Quantitative RT-PCR was performed using the SYBR Green Premix Pro Taq HS qPCR Kit (High Rox Plus (Accurate Biology), Hunan, China) and the ABI Step One Plus real-time PCR system. Total RNA was extracted from the samples (*G. biloba* episperm at three developmental stages) and synthesized into first-strand cDNA, and the reaction system containing cDNA was used for qRT-PCR. The parameters were set as pre-denaturation at 95 °C for 30 s, denaturation at 95 °C for 5 s, and annealing extension at 60 °C for 30 s for a total of 40 cycles, and a melting curve was plotted. Internal replicates were included, and three biological replicates were performed for all samples. Gene expression quantification was performed via the 2^−△△Ct^ analytical method.

### 2.6. Gene Cloning and Vector Construction

The coding sequence (CDS) of *GbKCS7* was amplified from *G. biloba* episperm cDNA using gene-specific primer P1 and subsequently ligated into the pClone007 Versatile Simple Vector (Tsingke, Cat. 007VS) for downstream applications. The positive clones were sequenced to confirm the accuracy of the cloned fragments. The CDS sequence of the *GbKCS7* gene was cloned into the pCAMBIA1300 vector using a specific primer (P2) containing *BamH I* and *Xbal I* restriction sites, fused to the GFP reporter under the control of the CaMV35S promoter. The recombinant vector was named 35S: GbKCS7-GFP. The coding sequence was directionally inserted into the pYES2 expression vector through *BamH I* and *Xho I* restriction sites engineered into primer P3, generating the recombinant construct designated pYES2-GbKCS7. Primer sequences are enumerated in the Supplementary Material (Table S1).

### 2.7. Subcellular Localization of GbKCS7 Gene

The *Agrobacterium tumefaciens* strain GV3101 containing the 35S: GbKCS7-GFP or 35S: GFP vector was infiltrated into *N. benthamiana* leaves. The GFP fluorescence signal was detected using a fluorescence microscope (Carl Zeiss, Oberkochen, Germany). The DsRed protein, connected with AtPIP2A (a cellular membrane aquaporin), was co-expressed with 35S: GbKCS7-GFP or 35S: GFP in *N. benthamiana* leaves [35].

### 2.8. Ectopic Expression of GbKCS7 Gene in Yeast

Plasmids pYES2-GbKCS7 and pYES2 were transformed into yeast (INVscI)-competent cells using Gene Pulser Xcell (BIO-RAD, Alfred Nobel Drive Hercules, CA, USA). Transformed yeast cells were cultured in a synthetic complete medium lacking uracil (SC/-Ura) containing 2% (*w*/*v*) glucose, followed by induction in SC/-Ura medium supplemented with 2% (*w*/*v*) galactose and 2% (*w*/*v*) raffinose for 24 h.

### 2.9. Generation of GbKCS7 Overexpression Lines in Arabidopsis

The Arabidopsis plants were transformed with the 35S: GbKCS7-GFP vector via floral dipping [36]. Homozygous T3 transgenic lines were identified over three consecutive generations through hygromycin (25 mg L^−1^) resistance screening on Murashige and Skoog (MS) solid medium. The genomic DNA of resistant seedlings was used as a template and verified by PCR using a specific primer (P16). The primer (P16) was designed from a 750 bp sequence between the CDS of the *GbKCS7* and GFP sequence. Quantitative RT-PCR analysis of *GbKCS7* expression in positive transgenic plants utilized *AtACT2* as a control gene and primers P11 and P17. Primer sequences are listed in the Supplementary Material (Table S1).

### 2.10. Extraction and GC-MS-Based Analysis of Fatty Acid Composition

The methods for extracting and analyzing fatty acids were adapted from previous research with minor adjustments [21]. A suitable volume of yeast suspension (OD600 = 1.4) was taken, and fatty acids were extracted from the yeast. The appropriate amount of Arabidopsis leaves was weighed and the fatty acids were extracted from them. An internal standard of 5 μl nonacosanoic acid (10 mg/mL) was incorporated. The fatty acid profile was determined via gas chromatography–mass spectrometry (GC-MS) with the specified equipment: (1) a Trace 1300 gas chromatograph (Thermo Fisher) with TG-FAME column (550 m × 0.25 mm × 0.20 μm), (2) helium carrier gas at constant flow (0.63 mL/min), and (3) an ISQ 7000 mass detector (Thermo Fisher) with EI ionization source The content of fatty acids was relatively quantified according to the intensity of the peaks related to the content of nonacosanoic acid.

### 2.11. Statistical Analysis

Each measurement involved three separate biological replicates. Specific primers were engineered employing Primer 5.0 software. Data processing was carried out using Microsoft Excel 2021 (Redmond, WA, USA). The statistical analysis for the *t*-test was performed using SPSS 26.0 (IBM, New York, NY, USA), with a significance threshold set at *p* < 0.05. Data from three independent experiments were analyzed and presented as mean values ± standard deviation using GraphPad Prism version 9.0 for graphical representation. Adobe Illustrator(Adobe Inc., Mountain View, CA, USA) was utilized to enhance and refine the images.

## 3. Results

### 3.1. Variation of C16:1 and C18:1 Content in G. biloba Episperm at Diverse Ontogenetic Phases

**Figure 1:**
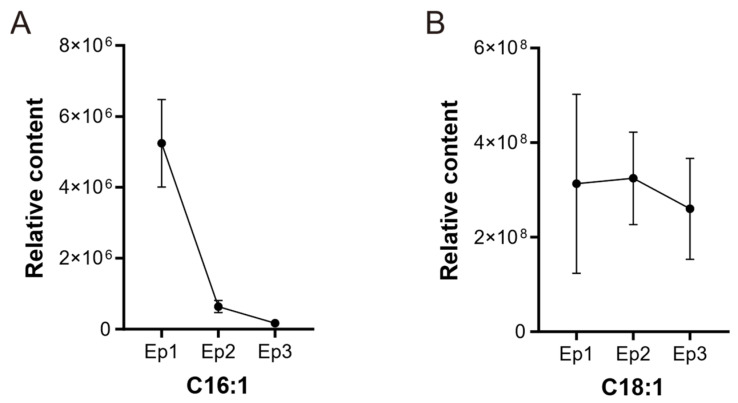
The relative content of two fatty acids during distinct developmental phases of *G. biloba* episperm. (**A**) The relative content of C16:1. (**B**) The relative content of C18:1.

The relative contents of C16:1 and C18:1 were analyzed based on metabolomic data of *G. biloba* episperm at different growth periods. Along with the development of episperm, the relative content of C16:1 gradually decreased from 5.2 × 10^6^ to 1.6 × 10^6^. The range of C18:1 content was between 2.6 × 10^8^ and 3.2 × 10^8^ and showed a downward trend at the Ep3 stage (Figure 1).

### 3.2. Identification of the GbKCS Genes and Their Location in G. biloba Chromosomes

**Figure 2:**
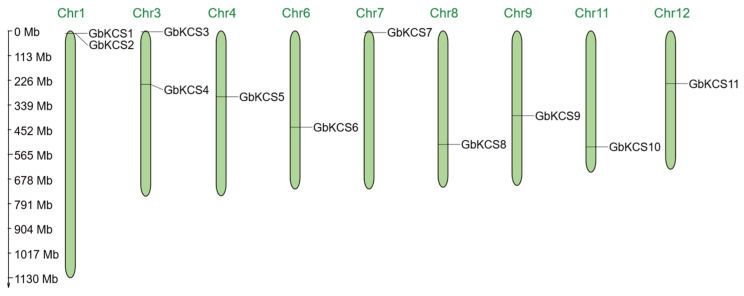
Chromosomal localization of the *GbKCS* genes. The top margin of every green vertical bar contained its assigned chromosome number designation, which reflected the approximate chromosomal locations of individual *GbKCS* genes across the genome.

In silico analysis revealed the presence of 11 *KCS* gene family members in the *G. biloba* genomic sequence, and they were evenly spread on 9 chromosomes (Figure 2). Chromosome 1 (Chr1) and Chr3 each possess two *GbKCS* genes, and the remaining 7 chromosomes each possess one *GbKCS* gene. The identified *GbKCS* genes were successively named from *GbKCS1* to *GbKCS11* according to their position on the chromosome.

The CDS length of the 11 *GbKCS* genes ranges from 1377 bp (*GbKCS1*) to 1827 bp (*GbKCS5*). The GbKCS proteins exhibited variable physicochemical parameters (Table S2), with GbKCS5 (608 amino acids) and GbKCS1 (458 amino acids) showing the maximum and minimum lengths, correspondingly. The theoretical isoelectric point of these KCS proteins ranges from 7.82 (GbKCS9) to 9.36 (GbKCS1), all of which are greater than 7.0, indicating that they are basic proteins. The average grand value of hydropathicity (GRAVY) ranges from −1.150 (GbKCS6) to 0.019 (GbKCS2), suggesting that most GbKCSs are hydrophilic proteins.

### 3.3. Phylogenetic Analysis of GbKCS Genes and Their Homologues from Other Species

Evolutionary relationships between *G. biloba* and other species were inferred from comparative analysis of 11 GbKCS and 21 AtKCS proteins, including sequence alignment and domain conservation assessment. These analyses revealed that all identified proteins possessed two characteristic conserved domains: FAE1_CUT1_RppA and ACP_syn_III_C (Supplementary Figure S1). These domains exhibited remarkable sequence conservation across all examined KCS proteins, suggesting their critical functional importance in the enzymatic activity. In addition, the C-terminus of GbKCS proteins is highly conserved, whereas the N-terminus is more flexible.

**Figure 3:**
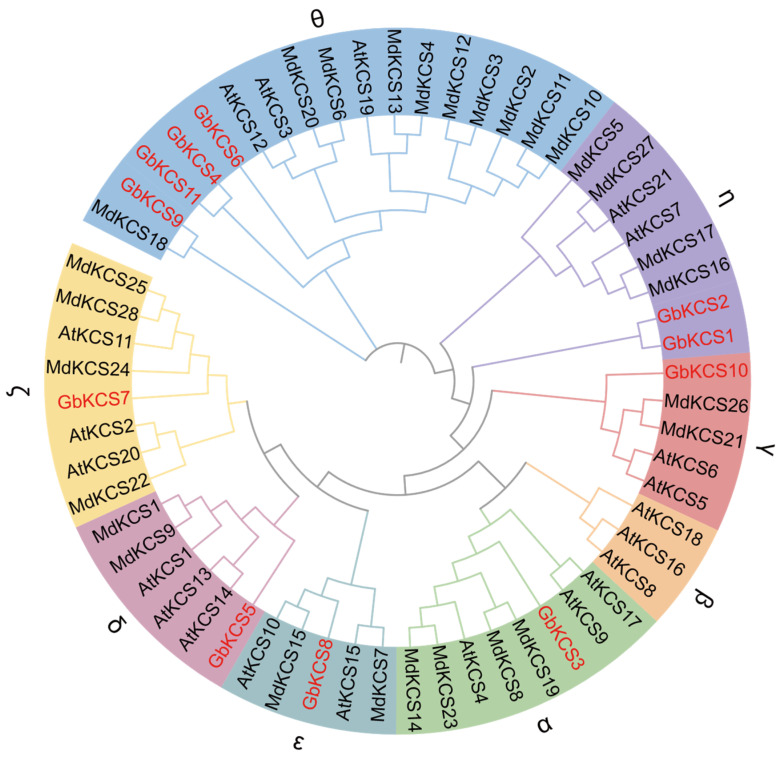
Phylogenetic tree of the *KCS* gene family in *A. thaliana*, *G. biloba*, and *M. domestica*. The evolutionary tree was generated from full-length protein sequences employing maximum likelihood algorithms. Eight subgroups were assigned in disparate shades, while *G. biloba KCS* family members were highlighted in red for immediate identification.

A total of 60 KCS protein sequences derived from *A. thaliana* (AtKCS), *M. domestica* (MdKCS), and *G. biloba* (GbKCS) were used to assess the phylogenetic relationship (Figure 3). A total of 21 AtKCSs were segregated into four main groups (KCS1-like, FAE1-like, FDH-like, and CER6) [32,37]. They were further classified into eight subgroups, including α, β, γ, δ, ζ, ε, η, and θ, based on protein homology relationships and structural features [38,39]. The θ subgroup contains the most members of GbKCSs and MdKCSs. The α, γ, δ, ζ, and ε subgroups each contained 1 GbKCS protein. Additionally, the η subgroup contained 2 GbKCS proteins (GbKCS1 and GbKCS2), while no GbKCS and MdKCS are classified into the β subgroup.

### 3.4. Conserved Motifs and Gene Structure of the GbKCS Genes

**Figure 4:**
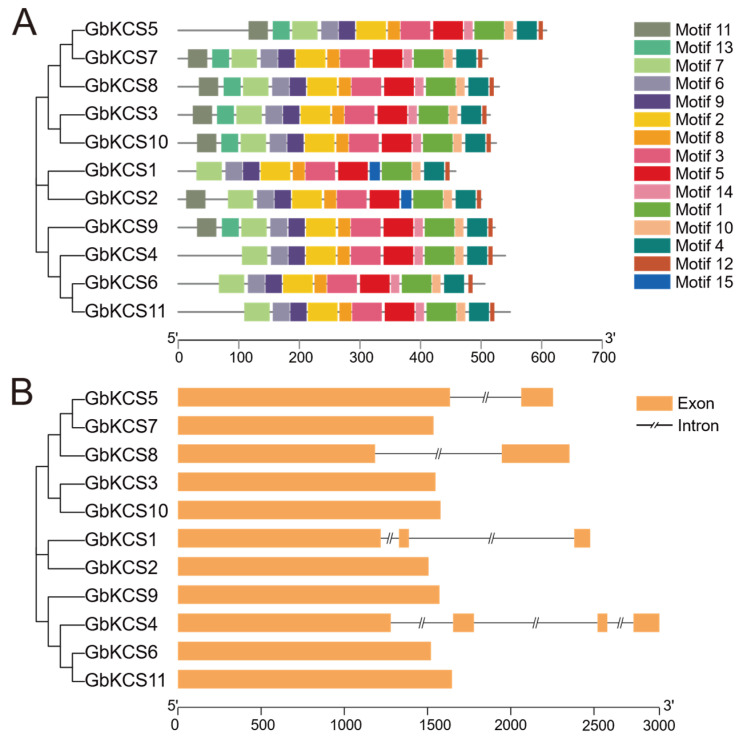
The conserved motifs and gene structure analysis of *GbKCS* genes. (**A**) The conserved motifs of GbKCS proteins. (**B**) The gene structure of *GbKCS*. Different motifs are shown as boxes with different colors. The yellow bar represents exons (expressed regions).

Most GbKCS proteins have identical type and number of conserved motifs (Figure 4A). Four GbKCS proteins (GbKCS1, GbKCS4, GbKCS6, and GbKCS11) contain 12 conserved motifs, and GbKCS2 contains 13 conserved motifs, while the remaining 6 proteins contain 14 conserved motifs. All identified proteins consistently contained motifs 1 through 10 and motif 12 in their sequences. GbKCS proteins of the same subgroup exhibit similar patterns of conserved motifs. For example, GbKCS4, GbKCS6, and GbKCS11 have the same type and number of conserved motifs. While only GbKCS1 and GbKCS2 contain the motif 15.

Analysis of the *GbKCS* gene structure established that seven *GbKCS* genes were intronless. *GbKCS5* and *GbKCS8*, which belong to the δ and ε subgroups, respectively, each contained one intron. *GbKCS1*, which is part of the η subgroup, contained two introns. In contrast, *GbKCS4*, which belongs to the θ subgroup, contained three introns and four exons (Figure 4B).

### 3.5. Cis-Acting Element Analysis of the GbKCS Gene Promoters

**Figure 5:**
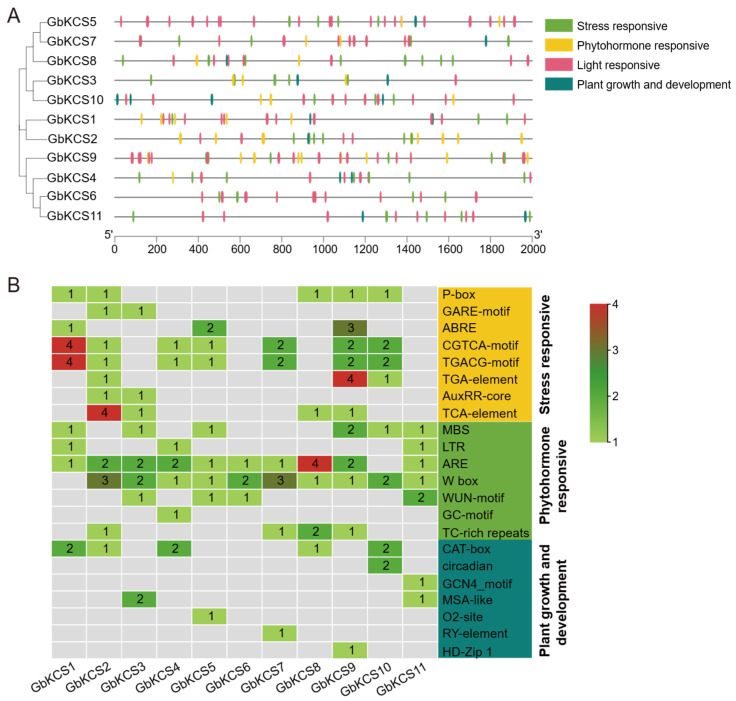
The cis-acting elements analysis in *GbKCS* genes. (**A**) Location of the four classifications of cis-acting elements in the promoter region. (**B**) Name and number of plant growth and development, stress response, and phytohormone response cis-acting elements of *GbKCS* genes. Distinct color codes were assigned to categorize various classes of cis-regulatory elements.

The cis-acting element prediction information of the 2000 bp promoter sequence upstream of *GbKCS* demonstrated that there were four types of elements in this segment: plant hormone signaling, stress adaptation, plant growth and development, and light response (Figure 5A). The MeJA response elements (CGTCA-motif and TGACG-motif) were found in 7 *GbKCS* genes (*GbKCS1*, *GbKCS2*, *GbKCS4*, *GbKCS5*, *GbKCS7*, *GbKCS9*, and *GbKCS10*) (Figure 5B). The gibberellin response elements P-box and GARE-motif were identified in 5 and 2 *GbKCS* genes, respectively. In addition, phytohormone response elements such as abscisic acid (ABRE), auxin (AuxRR-core and TGA-element), and salicylic acid (TCA-element) were found. These discoveries implied that most of the *GbKCS* genes were potentially under the control of a variety of phytohormones, the modulation of the expression of which methyl jasmonate (MeJA) and gibberellin may have been vital. Furthermore, stress response elements were detected in the promoter portions of the *GbKCS* genes. Including MBS, LTR, ARE, Wbox, and WUN-motif, these cis-elements were associated with defense and stress processes such as drought induction, low temperature response, anerobic induction, pathogen trauma response, and mechanical injury response. The promoter regions of *GbKCS* genes also contained cis-elements associated with plant growth, development (CAT-box, MSA-like, and circadian), and light response.

### 3.6. Expression Pattern of the GbKCS Genes

**Figure 6:**
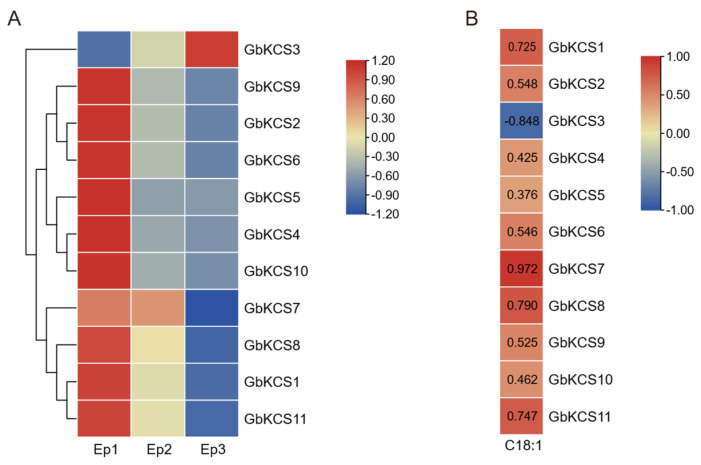
Heatmap of *GbKCS* gene expression in *G. biloba* episperm and correlation analysis with C18:1 content. (**A**) Heatmap of the *GbKCS* genes’ expression in *G. biloba* episperm at different developmental stages. (**B**) Heatmap of Pearson correlation between C18:1 and *GbKCS* genes in *G. biloba* episperm, with red and blue color codes denoting high and low correlation values, individually.

In light of the clustering of expression patterns, the 11 *GbKCS* genes were categorized into upregulated and downregulated expression patterns (Figure 6A). During episperm development, all *GbKCS* genes were downregulated, except for GbKCS3. Notably, most *GbKCS* genes were highly expressed at the Ep1 stage, except for *GbKCS7*, which showed predominant expression at both the Ep1 (FPKM = 80.18) and Ep2 (FPKM = 76.36) stages. Additionally, qRT-PCR validation of *GbKCS* expression profiles demonstrated strong concordance with the FPKM values, verifying the reliability of our transcriptomic data (Supplemental Figure S2).

Pearson correlation analysis was performed between C18:1 content and the expression levels of the *GbKCS* genes to explore the key *GbKCS* genes involved in C18:1 biosynthesis. The outcomes revealed that the C18:1 content in the episperm was positively associated with the expression of *GbKCS*, except for *GbKCS3* (Figure 6B). Among these, the correlation coefficient between the C18:1 content and expression of *GbKCS7* was 0.972, indicating a significant correlation between them. Thus, we hypothesized that *GbKCS7* likely participates in C18:1 biosynthesis in *G. biloba* episperm.

### 3.7. Cloning and Subcellular Localization of GbKCS7 Gene

**Figure 7:**
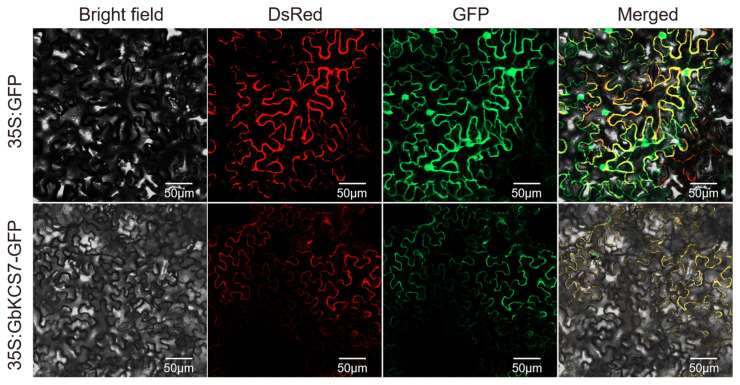
Subcellular localization of the *GbKCS7* gene. 35S: GFP and 35S: GbKCS7-GFP represent the control and *GbKCS7* expression vectors, respectively. They were co-expressed with DsRed fused with plasma membrane marker protein in *N. benthamiana* leaf epidermal cells. Scale bar = 50 μm.

Sequence analysis predicted *GbKCS7* to encode 511 amino acids, with its full-length CDS measuring 1536 base pairs (Figure S3). The outcomes of subcellular localization indicate that the GbKCS7-GFP was highly abundant in the plasma membrane (Figure 7). Thus, the protein encoded by the *GbKCS7* gene functions in the plasma membrane.

### 3.8. Heterologous Expression of GbKCS7 Gene and Analysis of Fatty Acid Composition in Yeast

**Figure 8:**
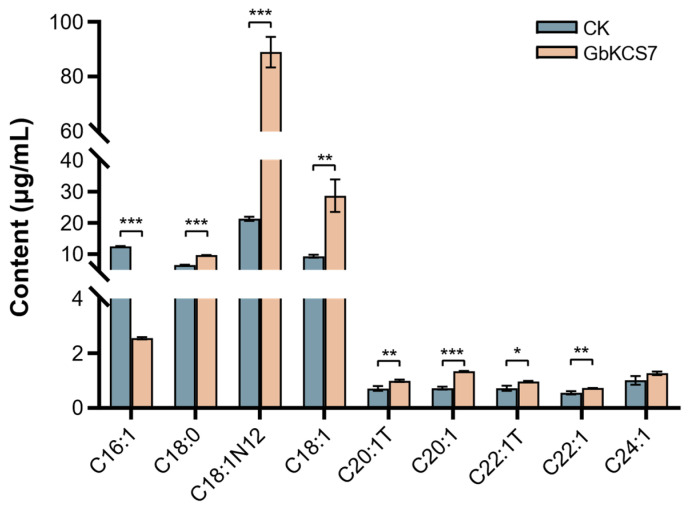
Changes in the fatty acid composition of the yeast by the induction of *GbKCS7*. Data represent mean ± standard error (SE) of three biological replicates. The *, **, and *** show significance at *p* < 0.05, *p* < 0.01 and *p* < 0.001, respectively, among control and overexpression (*GbKCS7*) groups according to Student’s *t*-test.

The pYES2-GbKCS7 vector was transformed into yeast (INVscI) for ectopic expression, and the total fatty acids in transformed yeast cells were extracted for qualitative and quantitative analysis using GC-MS. The outcomes revealed that while the overexpression of *GbKCS7* did not affect the types of fatty acids in yeast cells, it did alter the content of certain fatty acids (Figure 8 and Figure S4). Specifically, the C16:1 content was significantly reduced to 20.42% of that in the control group (CK) due to *GbKCS7*. Conversely, compared to CK, the levels of C18:1N12 and C18:1 rose by 3.18- and 2.07-fold, respectively. Moreover, the contents of C18:0, C20:1, and C22:1 also grew by 48%, 85%, and 34%, separately. These findings suggest that *GbKCS7* primarily catalyzes the extension process from C16:1 to C18:1 and accelerates the subsequent biosynthesis of C18–C22 fatty acids.

### 3.9. Overexpression of GbKCS7 Gene and Analysis of Fatty Acid Composition in Arabidopsis

The 35S: GbKCS7-GFP vector was transformed into Arabidopsis, and aggregate 5 homozygous transgenic lineages were confirmed by PCR with specific primers (Figure S5). Following this, qRT-PCR was applied to measure the relative expression of *GbKCS7* between different lines (Figure S6). *GbKCS7-OE-1* and *GbKCS7-OE-2*, two lines with the highest expression level of *GbKCS7*, were picked for further examination. Morphological observation demonstrates that overexpression of the *GbKCS7* gene exerted no significant effect on the growth of transgenic lines (Figure S6).

**Figure 9:**
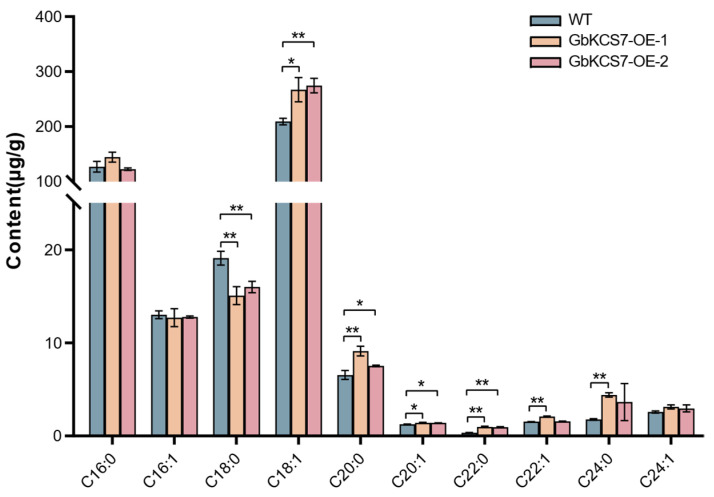
Fatty acid composition of Arabidopsis plants. Data represent mean ± standard error (SE) of three biological replicates. The * and ** show significance at *p* < 0.05 and *p* < 0.01, respectively, among WT and *GbKCS7-OE* Arabidopsis according to Student’s *t*-test.

The results of the fatty acid composition we measured in the leaves of transgenic Arabidopsis and WT showed that C18:1 in the GbKCS7-OE-1 and GbKCS7-OE-2 lines increased by 27.70% and 31.43%, respectively, compared with that in WT (Figure 9). This result further validates our speculation that the *GbKCS7* gene specially catalyzes the extension of C16:1 to produce C18:1. In addition, in the *GbKCS7-OE-1* and *GbKCS7-OE-2*, C20:1 was increased by 12.96% and 11.87%, C20:0 was increased by 39.44% and 14.98%, and C22:0 was increased by 180.36% and 170.94%, respectively.

## 4. Discussion

In plants, the *KCS* genes are comprehensively associated with the growth and development processes. For instance, in cotton (*Gossypium barbadense*), the *GbKCS* genes are closely associated with fiber elongation [40,41]. The *VvKCS* genes have a potential role in grapevine (*Vitis vinifera* L.) pollen wall formation [42]. Beyond involvement in growth and development, *KCS* genes are widely involved in plant resistance in response to both biological and environmental stressors. For example, the heterologous expression of *CsKCS6* in Arabidopsis promotes the accumulation of VLCFAs, which then improves the tolerance of transgenic plants to drought [43]. *VvKCS11* (grapevine L.) and *CqKCS2B.1* (*Chenopodium quinoa*) improve the resistance of transgenic Arabidopsis to salt stress [44,45].

In this investigation, 11 *KCS* genes identified from *G. biloba* genomic and transcriptomic data that were variably expressed in the episperm all had two completely conserved domains (FAE1_CUT1_RppA and ACP_syn_III_C), which were typical of the *KCS* gene family. The FAE1_CUT1_RppA structural domain encompassed the protein’s active site and regions involved in substrate binding [46]. Meanwhile, in the context of both plant and bacterial systems, the ACP_syn_III_C structural domain was crucial for beginning the fatty acid synthesis enzyme process chain, facilitating the combination interaction between acetyl-CoA and malonyl ACP [47]. GbKCS proteins were weakly basic, and our analysis of the amino acid sequences of AtKCS and GbKCS proteins revealed that the N terminus of KCS proteins was less conserved. This finding aligned with observations in sorghum (*Sorghum bicolor*) [48], leading us to hypothesize that this might be linked to variations in substrate-binding recognition, despite the presence of conservation. The multispecies phylogenetic tree revealed that KCS proteins were separated into eight subfamilies, aligning with the findings in Arabidopsis [38]. Conversely, the 11 *GbKCS* gene-encoded proteins were organized into seven subclades, with any GbKCS proteins found in the FAE-like (β) subclade, similar to those in peanut and passion fruit [13,17].

The identification and analysis of cis-acting elements within gene promoter regions were instrumental in predicting their potential functions and transcriptional regulation mechanisms. Previous investigations had indicated that *GbKCS* genes might be a key factor in plant hormone signal transduction and stress response [49]. In this investigation, we identified numerous cis-acting elements upstream of the *GbKCS* gene, including methyl jasmonate elements (CGTCA-motif, TGACG-motif), abscisic acid response element (ABRE), MYB binding site (MBS), and anaerobic response element (ARE). In sweet cherry (*Prunus avium* L.), exogenous MeJA significantly increased the expression levels of *KCS1* and *KCS6* and increased the waxy component in the epidermis of ripe fruit [50]. Cuticular wax is an important barrier that reduces fruit water loss and resists environmental stress and was essential for the proper development of fruits and seeds [29].

The distinct expression patterns of *GbKCS* genes in *G. biloba* episperm suggested that they played essential roles at various developmental stages. Specifically, the expression level of *GbKCS3* gradually elevated as the exocarp matured, whereas that of *GbKCS7* was highly expressed during Ep1 and Ep2 but was downregulated at Ep3. In citrus, *Cs7g13310* and *Cs7g28170* were highly expressed in the exocarp of fruits during early developmental stages, whereas *CsKCS2* (*Cs6g02360*) and *CsKCS11* (*Cs4g17260*) were prominently expressed during fruit ripening stages, indicating their potential roles in wax synthesis in both young and ripe fruits [19]. Based on these observations, we hypothesized that *GbKCS7* was crucial for fatty acid synthesis and accumulation during the pre-expansion stage of exocarp development. Further expression studies in yeast and Arabidopsis systems were necessary to validate its functions.

Heterologous expression in yeast is a common method to study the substrate specificity and catalytic function of proteins encoded by *KCS* genes in plants [22]. Several studies have shown that *KCS* genes in multiple species, such as Arabidopsis and rape, can exhibit catalytic activity in wild-type or Δ*elo3* yeast [20,51,52]. Functional characterization of *HaKCS1* through heterologous expression in *Helianthus annuus* in wild-type yeast increased the accumulation of VLCFAs, specifically C22:0, C24:0, and C26:0 [53]. Using the same method to discover the catalytic function of five *ZmKCS* genes in maize (*Zea mays*), *ZmKCS2* produced C20 and C22 VLCFAs; *ZmKCS4*, *ZmKCS11*, and *ZmKCS20* produced C24; and *ZmKCS15* is involved in the synthesis of C26 [54]. In this study, the *GbKCS7* gene was heterologously expressed in yeast, mainly manifested by the decrease in C16:1 and the increase in C18:1, C20:1, and C22:1. This result is similar to that of Batsale et al. [21], where *AtKCS1* extended the C16 fatty acid in yeast to C18 up to C22. It follows that both *GbKCS7* and *AtKCS1* seem to exhibit the ability to produce C18:1.

In Arabidopsis, the overexpression of *GbKCS7* resulted in a notable rise in the levels of C18:1 and C20:1, along with an increase in the content of saturated fatty acids such as C20:0 and C22:0. This proposes that *GbKCS7* is participating in the synthesis of C18:1 and extension of VLCFAs. However, the increase in saturated fatty acid content is not reflected in the yeast expression system, which may be due to the lack of a specific cofactor or a certain KCS protein interacting with *GbKCS7*, leading to functional differences from those shown in yeast. Some proteins in plants are cofactors for FAE to complete the carbon chain elongation process, for instance, the synthesis of VLCFAs requires the participation of CER2-like proteins in Arabidopsis and rice [55,56,57]. Moreover, it was found that homo- and hetero-dimerization can be formed between KCS2, KCS6, and KCS9 proteins in Arabidopsis, indicating that FAE may contain a variety of KCS and that KCS can form heterodimers [58]. The research of Huang et al. showed that KCS3 was able to bind to KCS6 to form a heterodimer, resulting in a decrease in KCS6 activity [59]. In both yeast and Arabidopsis, the outcomes of observing overexpression of *GbKCS* substantiated the findings from the early-stage combined metabolomic and transcriptomic analyses. This evidence underscores the pivotal role of *KCS7* in oleic acid accumulation and its significant impact on modifying VLCFA content.

Fatty acids C22 and C24 serve as crucial substrates in the synthesis of cuticle wax and aliphatic suberins, with the penetrability of the plant epidermis typically linked to the cuticular wax content. *KCS2*/*DAISY* and *KCS20* in Arabidopsis extend C20 to C22, and thus participate in the production and regulation of cuticle wax and aliphatic suberins [60]. Overexpression of the *CqKCS2B.1* gene from quinoa in Arabidopsis promoted the accumulation of C22 and C24 and increased the salt resistance of the plants [44]. *MdKCS2* and *CsKCS6* diminish the permeability of the Arabidopsis epidermis and enhance the tolerance of plants to abiotic stresses [43,61]. In this research, overexpression of *GbKCS7* resulted in an increased C22 content in Arabidopsis, which may enhance the plant’s resilience to abiotic stress. Nevertheless, to better inform genetic breeding efforts aimed at developing highly resistant *G. biloba* varieties, it is essential to further analyze its biological function concerning the cuticular wax content in overexpressed Arabidopsis.

## 5. Conclusions

In *G. biloba* episperm, we identified 11 *GbKCS* genes differentially expressed in this study, and their physicochemical properties, phylogeny, cis-acting elements, and expression pattern were evaluated to furnish new understanding of the gene family. A gene named *GbKCS7* was selected, and its function in fatty acid biosynthesis was further verified. Heterologous expression of *GbKCS7* increased the content of C18:1N12 and C18:1 in yeast by 3.18- and 2.07-fold, respectively, and this gene increased the content of C18:1 and VLCFAs in Arabidopsis and reduced the permeability of the leaf epidermis. Our findings demonstrate that *GbKCS7* plays an essential function in fatty acid synthesis regulation in *G. biloba* episperm and has a potential role in plant endurance to abiotic stress. This information provides a reference for genetic breeding with high resistance of *G. biloba* varieties.

## Data Availability

The transcriptome data of G. biloba episperm has been deposited as a BioProject under accession PRJNA1026889.

## Supplementary Materials

The following supporting information can be downloaded at: https://www.mdpi.com/article/10.3390/genes16070773/s1, Table S1. Primer sequences for PCR amplification and qRT-PCR, Table S2. Physiochemical properties of the *GbKCS* genes; Figure S1. Multi-sequence alignment and domain analysis of the AtKCS and GbKCS Proteins. Figure S2. Transcriptome FPKM expression data were verified by qRT-PCR. Figure S3. PCR electrophoresis diagram of *GbKCS7* genes. Figure S4. Heatmap of fatty acid content clustering in yeast overexpressing. Figure S5. Arabidopsis genomic DNA was used as a template for PCR validation. Figure S6. Relative expression levels of *GbKCS7* in overexpressed Arabidopsis plants. Figure S7. Phenotype of *GbKCS7* overexpressing Arabidopsis.
