# Supplementary material for: Genome-Wide Analysis of KCS Gene Family in Ginkgo biloba L. and Functional Identification of KCS7 in Oleic Acid Synthesis
Source: Genes (Basel). 2025 Jun 30;16(7):773. doi: 10.3390/genes16070773 (PMC12296010; doi:10.3390/genes16070773)
Supplement: Supplementary file 1 [file genes-16-00773-s001.zip › genes-3719647-supplementary.pdf]

Table S1. Primer sequences for PCR amplification and qRT-PCR

Primer ID	Primer Name	Sequences (5'-3')
P1	*GbKCS7-F*	ATGGAGGCTAAGAAGCCCGAT
	GbKCS7-R	TCAGATGTTCGAAACCCTAGGA
P2	*GbKCS7-M-F1*	CGGGATCCATGGAGGCTAAGAAGCCCGAT
	GbKCS7-M-R1	GCTCTAGATCAGATGTTCGAAACCCTAGGA
P3	*GbKCS7-M-F2*	CGGGATCCATGGAGGCTAAGAAGCCCGAT
	GbKCS7-M-R2	CGGGATCCATGGAGGCTAAGAAGCCCGAT
P4	*GAPDH-qP-F*	CAAGGACTCCAACACCTTACTC
	GAPDH-qP-R	CCGTGGATTCAACCACATACT
P5	*GbKCS1-qP-F*	CCAATGTGACCATCGCCCAGTATG
	GbKCS1-qP-R	TGCTTTGCCTCCAGAGTGAATGC
P6	*GbKCS2-qP-F*	TGGACCTCTGGTGCTGCCTTAC
	GbKCS2-qP-R	CACGGTCACATTGGTGCCTCTC
P7	*GbKCS3-qP-F*	ACAACAATGACGGCGATGGGATC
	GbKCS3-qP-R	GGTATGAGCAGCAGCGTGAGAAC
P8	*GbKCS4-qP-F*	AAGGAAGACGCAGAAGGCAACAC
	GbKCS4-qP-R	GCTGCTCGGACAAGGGAAGAAC
P9	*GbKCS5-qP-F*	AGAGGACGATACGGGAAGCATAGG
	GbKCS5-qP-R	TGCTACCAAGGTGCCAAAGAAGAG
P10	*GbKCS6-qP-F*	GTTTGGATCATACGCCTCCTGTGG
	GbKCS6-qP-R	TGTGTGCATCTTCCTGTCATCTGG
P11	*GbKCS7-qP-F*	TGCGTGTTACAAGCCTGTGGATG
	GbKCS7-qP-R	GTCTCCTCTCCCAACCCTGAGC
P12	*GbKCS8-qP-F*	GTCAGAGTGAGGAGGCGTTTGC
	GbKCS8-qP-R	TGAAAGCCAGCAGCAGCACAG
P13	*GbKCS9-qP-F*	AAGCCTACAGGGCAGCATTT
	GbKCS9-qP-R	TCCAAGTAAGGCAGCACCAG
P14	*GbKCS10-qP-F*	CCAAGGCATAGTCGGCACATCTC
	GbKCS10-qP-R	GGCAGAACCAGAGGACCCAGAG
P15	*GbKCS11-qP-F*	AGCCTCCAGAGTTCTGCCCATATC
	GbKCS11-qP-R	GCTCGACCACGAGGATGAATGC
P16	*GbKCS7-GFP-F*	TCCGAATGGCATATGGAGCC
	GbKCS7-GFP-R	TTCTGCTTGTCGGCCATGAT
P17	*AtACT2-F*	ATTCAGATGCCCAGAAGTCTTGT
	AtACT2-R	GAAACATTTTCTGTGAACGATTCC

Table S2. Physiochemical properties of the *GbKCS* genes

Gene name	Gene ID	Isoelectric point	Relative molecular mass	Instability index	Aliphatic index	Grand av erage of hydropat hicity	Amino aci d quantity	CDS length /bp	Subcellular Localization
GbKCS1	*Gb_06885*	9.36	51519.51	40.49	89.00	-0.114	458	1377	chloroplast
GbKCS2	*Gb_06884*	8.81	56315.13	37.98	97.11	0.019	501	1506	plasma membrane
GbKCS3	*Gb_20097*	9.12	57839.42	39.51	99.38	-0.010	515	1548	plasma membrane
GbKCS4	*Gb_17018*	8.49	60444.56	35.06	85.04	-0.131	540	1623	plasma membrane
GbKCS5	*Gb_10050*	8.98	68974.49	35.72	88.83	-0.066	608	1827	chloroplast
GbKCS6	*Gb_15941*	8.48	57515.70	43.31	90.69	-0.150	506	1521	plasma membrane
GbKCS7	*Gb_23820*	8.94	57913.44	34.85	94.21	-0.040	511	1536	plasma membrane
GbKCS8	*Gb_05331*	9.18	59949.70	41.94	90.13	-0.088	530	1593	plasma membrane
GbKCS9	*Gb_32625*	7.82	60044.89	44.62	87.82	-0.069	523	1572	plasma membrane
GbKCS10	*Gb_20857*	9.13	58992.70	40.43	95.22	0.007	525	1578	plasma membrane
GbKCS11	*Gb_08308*	8.40	62072.86	33.41	88.58	-0.026	548	1647	plasma membrane

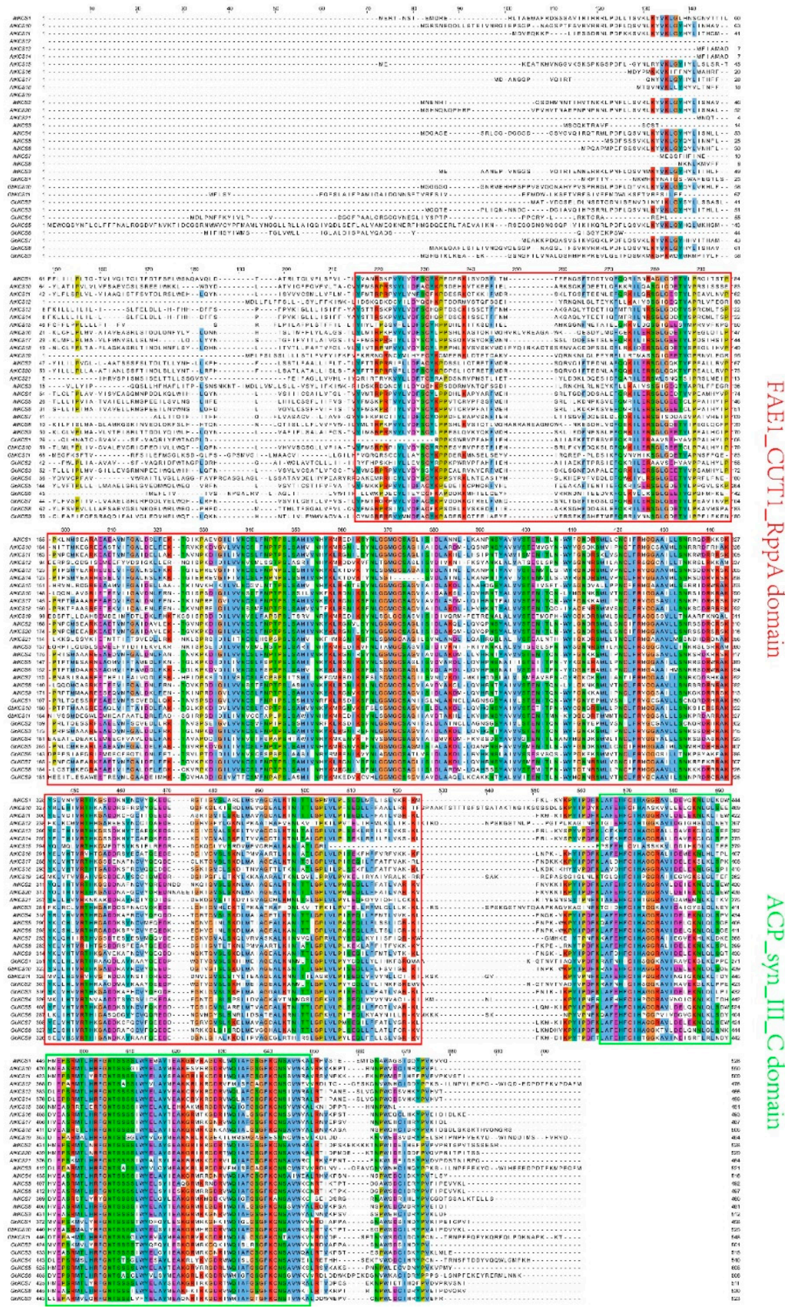

Figure S1. Multi-sequence alignment and domain analysis of the AtKCS and GbKCS Proteins. The red box represents the FAE1_CUT1_RppA domain, and the blue box represents the ACP_syn_III_C domain.

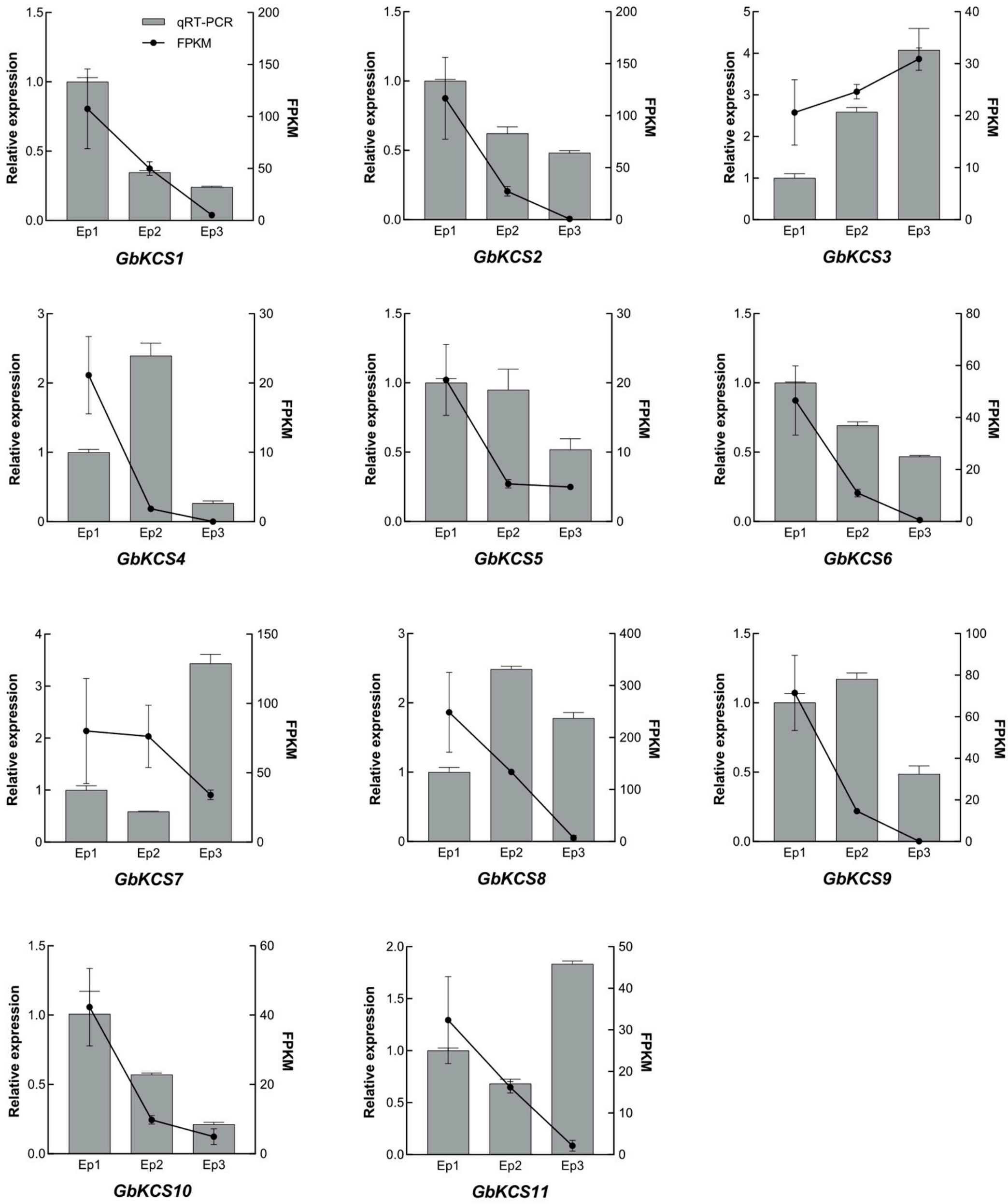

Figure S2. Transcriptome FPKM expression data were verified by qRT-PCR. The bar graph represents the qRT-PCR results for *GbKCS* genes in the outer seed skin at three different developmental periods, and the line plot represents the FPKM values for *GbKCS* genes in the transcriptome. The error bars in the plot represent the mean ± SD, the left Y axis represents the relative expression levels of qRT-PCR, and the right Y axis represents the FPKM values for the transcriptome data.

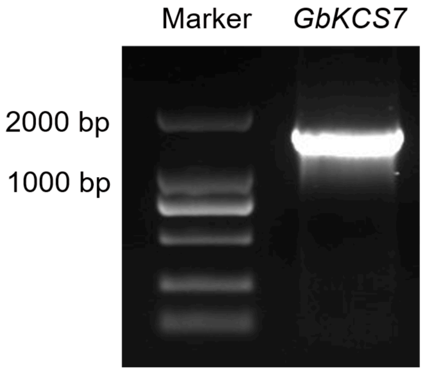

Figure S3. PCR electrophoresis diagram of *GbKCS7* genes.

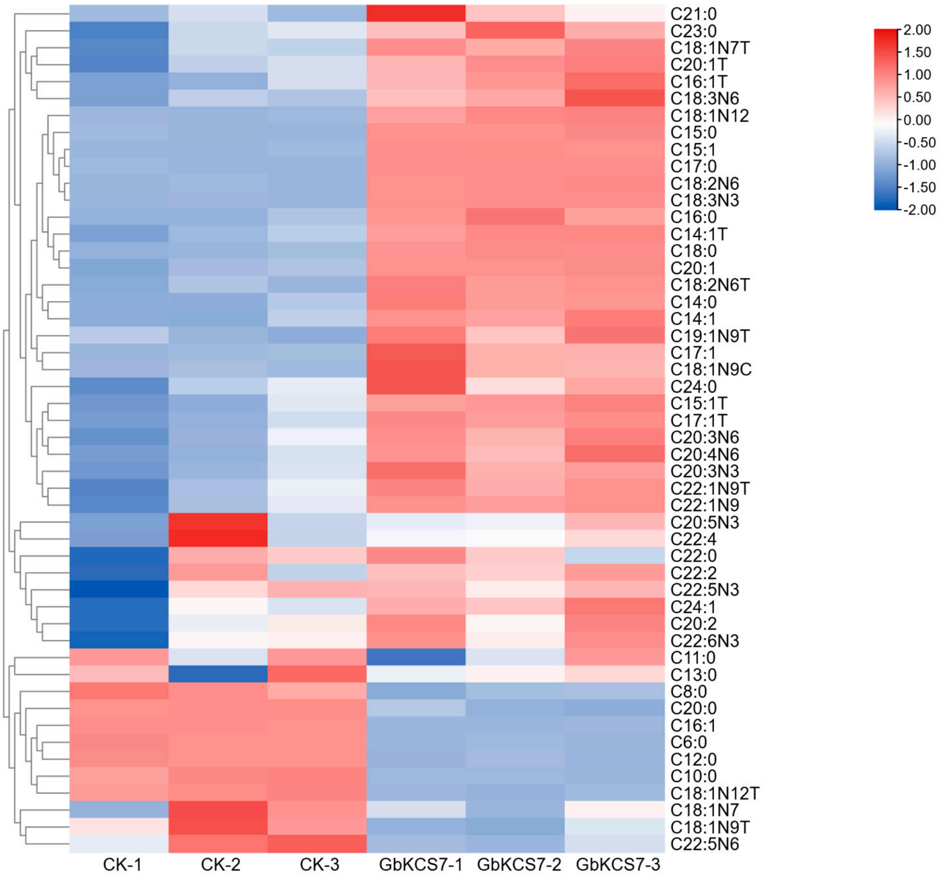

Figure S4. Heatmap of fatty acid content clustering in yeast overexpressing. Red represents high levels and blue represents low levels.

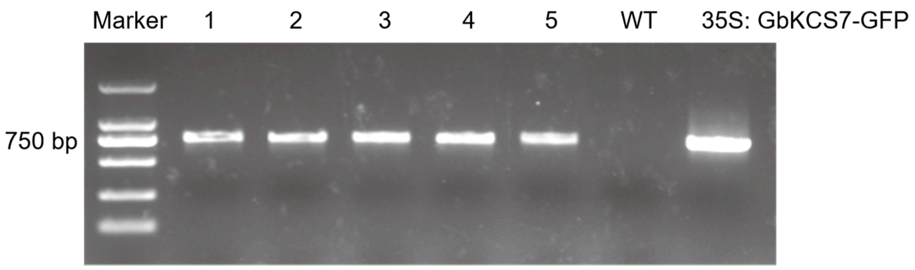

Figure S5. Arabidopsis genomic DNA was used as a template for PCR validation. 1 – 5 represent GbKCS7-OE-1 to GbKCS7-OE-5, respectively. WT represents wild-type Arabidopsis and the 35S:GbKCS7-GFP represents the GbKCS7 overexpression vector.

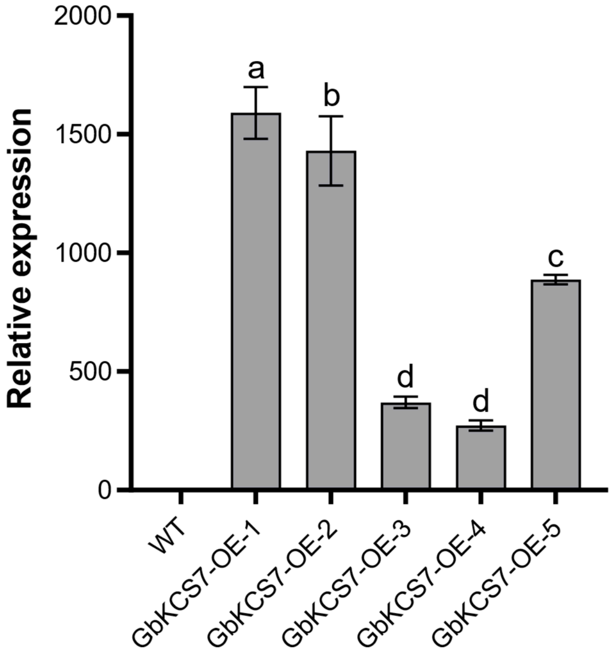

Figure S6. Relative expression levels of *GbKCS7* in overexpressed Arabidopsis plants.

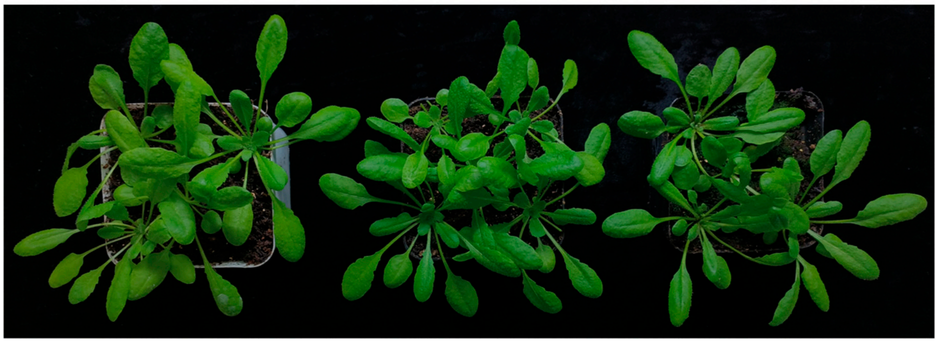

Figure S7. Phenotype of *GbKCS7* overexpressing Arabidopsis.
